# Testis development in the Japanese eel is affected by photic signals through melatonin secretion

**DOI:** 10.7717/peerj.12289

**Published:** 2021-10-15

**Authors:** Ji-Yeon Hyeon, Jun-Hwan Byun, Eun-Su Kim, Yoon-Seong Heo, Kodai Fukunaga, Shin-Kwon Kim, Satoshi Imamura, Se-Jae Kim, Akihiro Takemura, Sung-Pyo Hur

**Affiliations:** 1 Jeju Marine Research Center, Korea Institute of Ocean Science and Technology, Jeju, Republic of Korea; 2 Department of Biology, Jeju National University, Jeju, Republic of Korea; 3 Graduate School of Engineering and Science, University of the Ryukyus, Okinawa, Japan; 4 LED-Marine Biology Convergence Technology Research Center, Pukyong National University, Busan, Republic of Korea; 5 Aquaculture Research Division, National Institute of Fisheries Science, Busan, Republic of Korea; 6 Department of Chemistry, Biology and Marine Science, Faculty of Science, University of the Ryukyus, Okinawa, Japan; 7 Department of Ocean Science, University of Science and Technology, Daejeon, Republic of Korea; 8 Center for Strategic Research Project, University of the Ryukyus, Okinawa, Japan

**Keywords:** Japanese eel, Melatonin, Moonlight, Eel migration, Photoperiod, Photic signal

## Abstract

**Objective:**

According to reported spawning characteristics of Japanese eel, *Anguilla japonica*, which exhibit spawning and migration patterns that are synchronized with lunar cycles and photoperiod, we hypothesized that a close association exists between specific photic signals (daylight, daylength, and moonlight) and endocrinological regulation. Given the photic control in melatonin secretion, this hypothesis was tested by investigating whether melatonin signals act as mediators relaying photic signals during testis development in the eel.

**Methods:**

We examined changes in melatonin-secretion patterns using time-resolved fluorescence immunoassays in sexually immature and mature male Japanese eels under the condition of a new moon (NM) and a full moon (FM).

**Results:**

The eye and plasma melatonin levels exhibited a nocturnal pattern under a 12-h light: dark cycle (12L12D) or under constant darkness (DD), but not with constant light (LL). Eye melatonin levels were similar under the 12L12D and short-day (9L15D) conditions. In the long-day condition (15L9D), secreted plasma melatonin levels were stable, whereas short-day melatonin secretion began when darkness commenced. Sexual maturation began at 8 weeks following intraperitoneal injection of human chorionic gonadotropin (hCG), and NM exposure led to significantly higher eye and plasma melatonin levels compared with those detected under FM exposure.

## Introduction

Melatonin is an indoleamine hormone secreted during the night that regulates circadian rhythms, and that is mainly synthesized and secreted from the pineal gland and retinas. Once secreted from the pineal gland, melatonin is transported *via* the bloodstream and cerebrospinal fluid (CSF) to the central and peripheral tissues where it helps to regulate physiological, biochemical, and behavioral processes (Klein et al., 1997; Falcón et al., 2010). In contrast, melatonin secreted from the retinas influences retinomotor movements, neurotransmitter release, and neuronal electrical activity (Falcón et al., 2010; Besseau et al., 2006; Siu et al., 2006; Ping et al., 2008; Sauzet et al., 2008). Nonetheless, photo-sensors in the retinas and pineal gland sense photic cues through common photoreceptor cells, thereby directly participating in melatonin production (Cahill, Grace & Besharse, 1991; Falcón & Collin, 1991). Thus, in both the retinas and pineal gland, photic signals are involved in light sensing and in melatonin production and secretion (Iigo et al., 1994; Iigo et al., 2007; Ekström & Meissl, 1997; Falcón, 1999). However, information regarding mechanisms that regulate melatonin secretion from the eye induced by a photic signal remains insufficient.

Many animals sense photic cues that are used in physiological processes (Bromage, Porter & Randall, 2001). Currently, the known key aspects of photic cues include exposure time (duration) of daylight, intensity, and spectrum. Photoperiod changes occur periodically and predictably, whereas the light quality is more difficult to predict, and different organisms show varying degrees of photo-sensitivity depending on ecological conditions (Mazurais et al., 2000; Migaud et al., 2006). Effects of photoperiodic changes can be described for species inhabiting a temperate zone; however, such descriptions are limited for sub-tropical and tropical zones. However, tropical fish are considered to use relatively invariable photic signals and have different photo-sensing mechanisms from those of fish inhabiting temperate zones. Previous studies indicated that the moonlight intensity influences gonadal development and gamete release (Takemura et al., 2006; Takemura et al., 2004), along with melatonin secretion from the pineal gland and retinas (Takemura et al., 2006; Rahman et al., 2004) in goldlined spinefoot (*Siganus guttatus*) residing in sub-tropical and tropical zones. Thus, the photoperiod and water temperature function as a zeitgeber that synchronizes the reproductive rhythms of species living in aquatic environments in regions with relatively little variability.

Japanese eel (*Anguilla japonica*) has a catadromous life cycle. Glass eels that transform from the leptocephalus stage live in regions of northeast Asia after migrating to fresh water areas where they spend most of their lives (approximately 5–17 years) (Kotake et al., 2007). Yellow eels at the sexually immature stage also show locomotor behaviors based on the lunar cycle (Baras et al., 1998). Japanese silver eels begin spawning migration between September and November when puberty is initiated (Tsukamoto, 2009). Anguillids are nocturnal species whose locomotor activities increase during the night (Japanese eel: Aoyama et al., 2002; European eel: Tesch, 1978; Tesch, 1989; American eel *A. rostrata*: Helfman et al., 1983; shortfinned eel *A. australis* and longfin eel *A. dieffenbachii*: Jellyman & Sykes, 2003). The spawning period is suggested to be regulated by the lunar cycle at a site close to the West Mariana Ridge where spawning occurs during the last days of the lunar month (Sudo & Tsukamoto, 2015; Tsukamoto et al., 2003).

Previous studies have illustrated the importance of environmental factors for various reproductive traits of anguillid species, such as the influence of photoperiod changes on ovarian development in European eels (Parmeggiani et al., 2015), the influence of temperature on the spawning performance of artificially matured Japanese eels (Dou et al., 2008), and the influence of swimming performance on ovarian development in European eels (Palstra et al., 2007). These findings suggest that environmental factors may also be closely correlated with endocrinological regulation in anguillids and further support the possibility of a close correlation between melatonin production and environmental cues. However, no study has yet described the mechanism whereby environmental information is sensed, or how it might regulate melatonin secretion in eel which are catadromous fish. A study of the endogenous melatonin system based on environmental information is important for understanding how external light signals are converted internally in anguillids.

We hypothesized that a close association exists between specific photic signals and physiological regulation, regarding previously reported spawning migration of eels (Tsukamoto et al., 2003). This hypothesis could be tested by determining whether melatonin signals act as mediators relaying photic information as endocrine signals. To achieve this purpose, the following parameters were investigated: (1) examined eye and plasma melatonin levels of male Japanese eels for 24 h under conditions of a 12-h light: dark cycle (12L12D), constant light (LL), and constant darkness (DD), (2) the pattern of melatonin secretion under different photoperiod (short-day; 9L15D, long-day; 15L9D condition), and (3) examined eye and plasma melatonin levels under natural moonlight conditions [NM] and full moon [FM]) between immature and mature in Japanese eel males were comparatively analyzed.

## Materials & Methods

### Animals and maintenance

The Japanese yellow eels (*n* = 234, 2-years old) used in this study were cultivated males (body weight: 280–405 g) obtained from a commercial source in Gwangju Prefecture of South Korea. The eels were reared in indoor circular tanks (1-metric ton capacity) at 20 ± 1 °C in the Lava-water Aquatic Animals Care Center (Jeju Techno-Park, Jeju, South Korea) with continuously running fresh water under the artificial 12L12D condition (light on at 07:00 h and light off at 19:00 h, 600 lx, PPFD = 10.0 µmol m^−2^s^−1^, *λ*p = 545 nm) or the artificial LL condition with a white light-emitting diode (LED) light (KRGB3, SS Light, Co., Seoul, South Korea). No food was given to the fish during the experiments. All experiments were conducted in compliance with the guidelines of the Institutional Animal Care and Experimental Committee of Jeju National University that approved the experimental protocol (No. 2016-0039).

### Experiment 1: Variations of eye and plasma melatonin levels under the 12L12D, LL, and DD conditions

To evaluate daily and circadian fluctuations in melatonin levels in the eye and plasma, fish (42 individuals per tank) were housed in three fresh water tanks (1-metric ton capacity) without a fish shelter under the 12L12D condition with a water temperature of 20 ± 1 °C. Following a 1-week acclimation period, the fish were reared for 3 days under the 12L12D (light on at 07:00 h and light off at 19:00 h) or LL condition with LED lights and a water temperature of 20 ± 1 °C. The light intensity at the water surface was adjusted to approximately 600 lx (10.0 µmol m^−2^s^−1^, *λ*p = 545 nm). Fish (5 individuals per sampling time from one tank) were also held under the DD condition for comparison. Fish were anesthetized with 150 mg L^−1^ MS-222 (Sigma-Aldrich, St. Louis, MO, USA) and decapitated at 4 h intervals beginning at clock time (CT) 9.

### Experiment 2: Variations in eye and plasma melatonin levels under short- and long-day conditions

To examine the effects of photoperiodic changes on melatonin levels in the eyes and blood, fish (24 per tank) were housed in two tanks (1-metric ton capacity) under the 12L12D condition with a water temperature of 20 ± 1 °C for 1 week. After the 1-week acclimation period, the photoperiod in the tanks was changed to the short-day (SD) condition (9L15D, light on at 06:00 h and light off at 17:00 h) or the long-day (LD) condition (15L9D, light on at 06:00 h and light off at 21:00 h) for 1 week. Fish (5 per sampling time from one tank) were anesthetized and decapitated at 4-h intervals beginning at CT9.

### Experiment 3: Moonlight experiments

**Figure 1:**
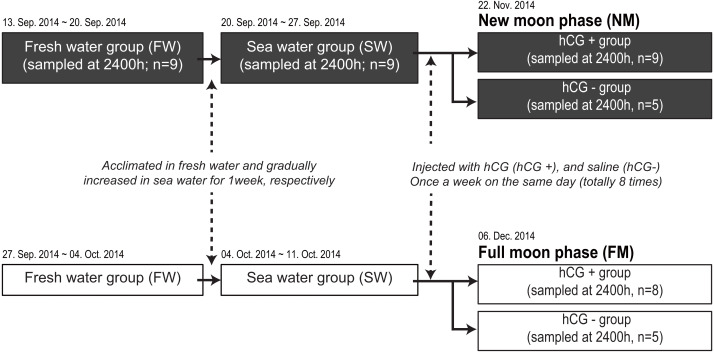
Experimental design for determining melatonin levels in ocular and plasma according to exposure to an NM or an FM. The solid and open squares represent the NM and FM groups, respectively. Following acclimation to fresh water for 1 week, the eels were gradually increased to a salinity approximating that of seawater for 1 week. After acclimation to seawater, the fish were transferred to two separate outdoor tanks for inducing sexual maturation. The fish were weighed and intraperitoneally injected with hCG (hCG+ group) or 0.6% saline (hCG–  group) once a week on the same day (totally eight times) until the sampling date (NM, November 22, 2014; FM, December 06, 2014).

We next compared the melatonin levels between the NM and FM periods according to testis development. A total of 71 fish were housed in an indoor tank with recirculating, aerated fresh water under the 12L12D condition at 20 ± 1 °C. Following acclimation for 1 week in fresh water, the salinity of each tank was gradually increased to the level in sea water for 1 week. After acclimation to sea water, the fish were transferred to two outdoor acryl tanks (3-metric ton capacity) to artificially induce testis development (Fig. 1). The rearing tanks were maintained under a natural photoperiod condition (approximately 11L13D) with recirculating water (20 ±1 °C), but without a cover on the tank, until the end of the experiment. After anesthesia and weighing, the fish were intraperitoneally injected with hCG at 1 IU/g body weight (hCG+ group) or injected with 0.6% NaCl (hCG– group) once a week for a total of 8 weeks. Following initial sample collection under the fresh water condition (*n* = 9) and after acclimation to sea water (*n* = 9), the fish were randomly taken from the tank at 2400 h. Sample collections around the NM (hCG+; *n* = 9, hCG–; *n* = 5) and the FM (hCG+; *n* = 8, hCG–; *n* = 5) periods were carried out on November 22, 2014 and December 6, 2014, respectively. On each sampling day, the fish were taken from the tank at 2400 h, anesthetized, and then sacrificed by decapitation in accordance with the guidelines mentioned above. After weighing, the eye on the left side was immediately collected, frozen in liquid nitrogen, and stored at −80 °C until analysis. Blood was collected from the caudal vein using a heparinized syringe, transferred into a microtube on ice, and centrifuged at 8,000× g for 10 min at 4 °C to obtain plasma. The collected plasma was stored at −80 °C until performing the time-resolved fluorescence immunoassay (TR-FIA) to estimate melatonin levels. The gonads were harvested from the body cavity and weighed. Gonad sections were fixed in Bouin’s solution for histological observation. All sample collections at 2400 h were carried out under dim-light conditions (1.5 lx, 0.0 µmol m^−2^s^−1^ at 670 nm) using a red-light LED module. The gonadosomatic index (GSI) and eye index (EI) (Pankhurst, 1982) were calculated as follows:

GSI = (gonadal mass/body mass) ×100

EI = {[(A + B)/4) 2 × *π*/TL (mm)]}

where A and B are the horizontal and vertical orbital diameter (mm), respectively.

### Melatonin measurements

The eyes on the left side were homogenized in 1 ml of 20 mM phosphate-buffered saline (pH 7.3) containing 0.5% bovine serum albumin (Sigma-Aldrich) and centrifuged for 15 min (10,000× g at 4 °C). Each supernatant was separated and stored on ice. Melatonin-containing fractions were extracted from each supernatant and plasma sample using Sep-Pak Vac C18 cartridges (Waters Corporation, Milford, MA, USA), which were activated with 1 ml of 100% methanol and then with 1 ml of distilled water (DW). After applying the supernatant/plasma (500 µl) and then DW (500 µl), each cartridge was washed twice with 1 ml of 10% methanol and then with hexane. Melatonin levels were measured by the TR-FIA as described by Takeuchi et al. (2014).

### Histological procedures

Testis samples were fixed in Bouin’s fluid. The fixed samples were dehydrated through an ethanol series, embedded in paraffin wax (Leica Biosystems, Richmond, IL, USA), and sectioned at 8 µm. Sections were stained with Mayer’s hematoxylin and eosin to study testis development.

### Data analysis

All statistical analyses were performed using GraphPad Prism 8.0 Software. Daily and circadian variations of melatonin levels were compared by one-way analysis of variance (ANOVA), followed by Tukey’s multiple-comparisons test. The GSI and EI values, and melatonin levels during artificially induced sex maturation under different moonlight conditions were compared by one-way ANOVA. Differences occurring under different photoperiod conditions (SD *vs.* LD) at each CT were determined by one-way ANOVA with an unpaired *t*-test. A *P*-value < 0.05 was considered to represent a statistically significant difference.

## Results

### Experiment 1: Variation of eye and plasma melatonin levels under the 12L12D, LL, and DD conditions

**Figure 2:**
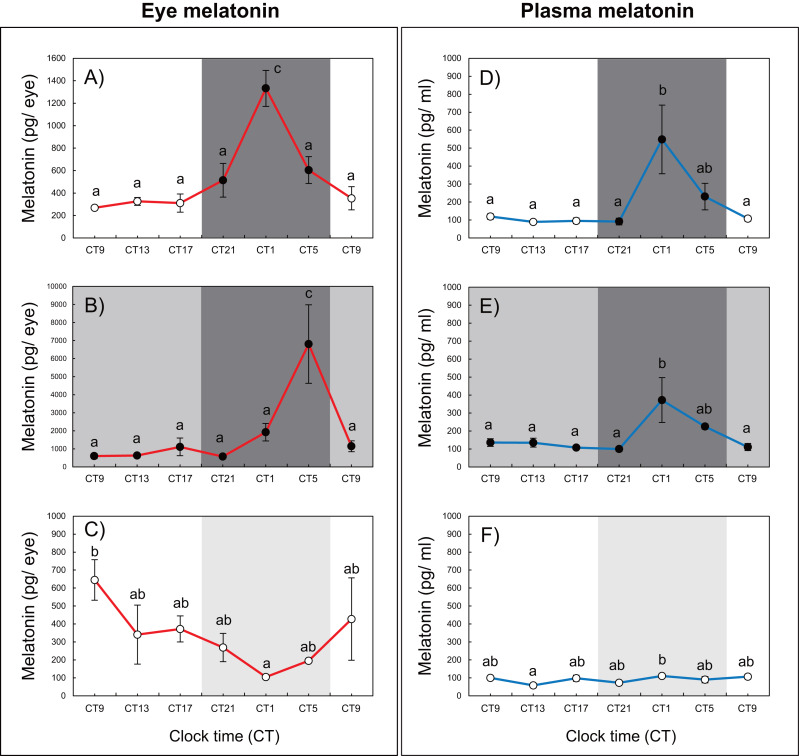
Variations of daily and circadian eye and plasma melatonin rhythms after 3 days of rearing under the 12L12D (eye; A, plasma; D), DD (eye; B, plasma; E), and LL (eye; C, plasma; F) conditions. The values shown for the melatonin levels represent the means ± standard errors of the mean (SEMs) (*n* = 5–6 fish per time point), where duplicate determinations were performed for each sampling time. The open and solid bars of each graph represent the scotophase and photophase, respectively. Significant differences between the means at each sampling time are indicated by different letters (one-way ANOVA, A; *F* = 12.22, *df* = 33, B; *F* = 6.41, *df* = 33, C; *F* = 2.456, *df* = 33, D; *F* = 5.345, *df* = 30, E; *F* = 4.583, *df* = 31, F; *F* = 2.702, *df* = 32, *P* <  0.05).

Variations in circadian levels of eye and plasma melatonin are shown in Fig. 2. A similar pattern was exhibited for both eye and plasma melatonin levels under the 12L12D and LL conditions. Both eye and plasma melatonin patterns under the 12L12D cycle showed clear day–night changes, with levels peaking at CT1 and decreasing from CT5 (Figs. 2A, 2D) (*P* <  0.05). In contrast, under the LL condition, eye melatonin levels were significantly higher at CT9 than at CT1 (Fig. 2C) (*P* < 0.05), and plasma melatonin levels were significantly higher at CT1 than at CT13 (Fig. 2F) (*P* < 0.05); however, both tissues showed lower melatonin levels under the LL condition compared with those measured under the 12L12D and DD conditions. Fundamentally, the patterns of eye and plasma melatonin levels in the 12L12D and DD conditions were similar: the levels of melatonin increased during scotophase and decreased at photophase. However, different peak times were exhibited under the DD condition in terms of the eye and plasma melatonin levels. The levels of eye melatonin peaked significantly at CT5 (Fig. 2B) (*P* < 0.01), whereas the levels of plasma melatonin peaked significantly at CT1 (Fig. 2E) (*P* < 0.05).

### Experiment 2: Variation in eye and plasma melatonin levels under SD and LD conditions

**Figure 3:**
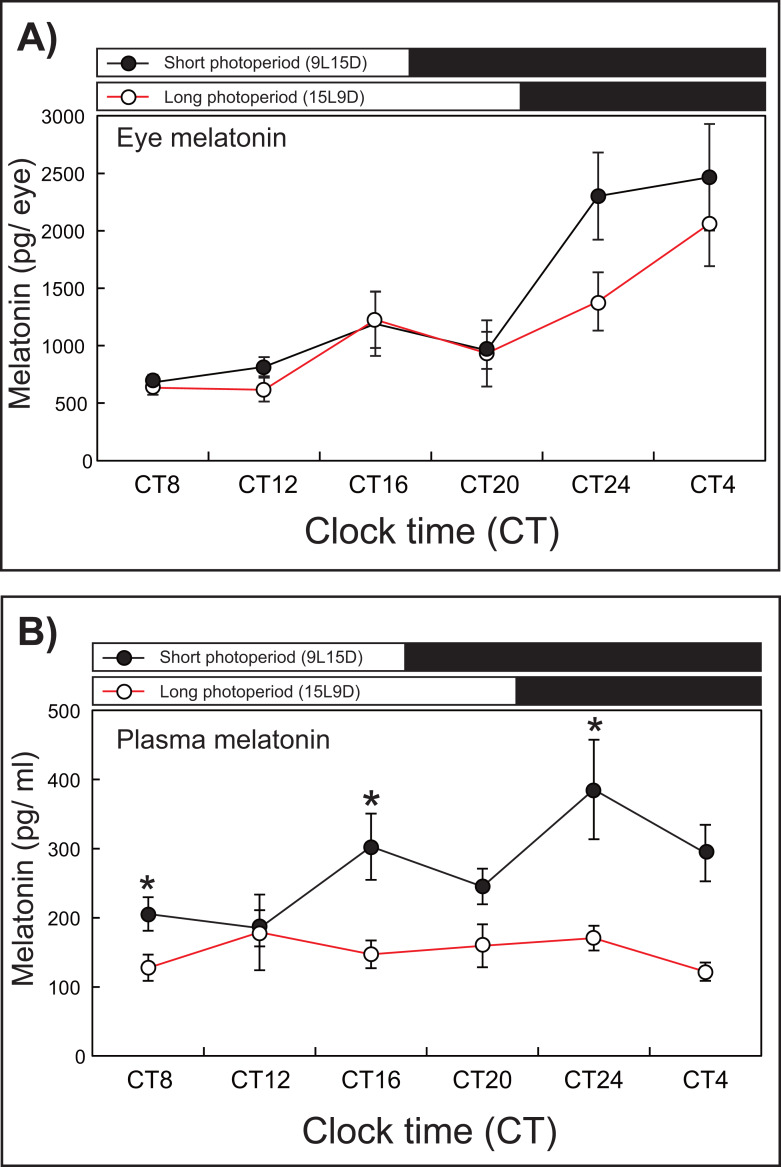
Photoperiodic changes of eye (A) and plasma (B) melatonin levels under a short-day condition (SD, 9L15D) and a long-day condition (LD, 15L9D). The values of the melatonin levels shown represent the means ± SEM (*n* = 5 per time point) of duplicate determinations for each sampling time. The solid and open circles indicate the SD and LD conditions, respectively. The open and solid bars at the top of each graph represent the scotophase and photophase, respectively. The asterisk indicates statistically different levels of melatonin observed between same sampling points (unpaired *t*-test, *P* < 0.05).

After 1 week of rearing, the eye melatonin levels under the SD condition increased significantly and peaked from CT24 to CT4, whereas these levels peaked at CT4 under the LD condition (15L9D) (Fig. 3). The daily plasma melatonin levels under the SD condition increased slightly after the lights were turned off (CT16) and peaked at CT24, whereas low daily plasma melatonin levels were maintained under the LD condition.

### Experiment 3: Influence of moonlight on testis development and melatonin levels

### Histological observations

**Figure 4:**
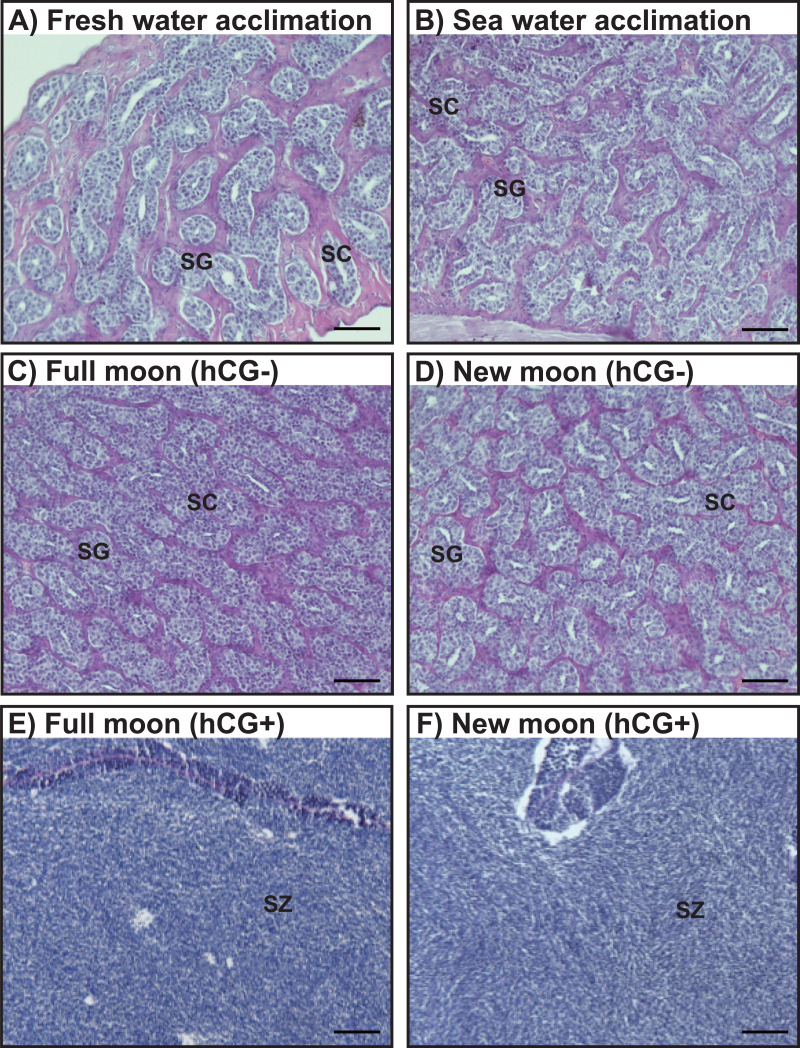
Histological observation of the testis development. Light-micrographs images of the testes from the groups acclimated to FW (A) and SW (B). After an 8 weeks treatment with hCG or saline, histological observations were performed to examine the testes from the FM (hCG–; C, hCG+; E) and NM (hCG–; D, hCG+; F) groups. The testes were sectioned after paraffin embedding and stained with hematoxylin and eosin.

Histological observations revealed that the testes of acclimated eels reared in fresh water (Fig. 4A) and sea water (Fig. 4B) were immature and contained both spermatogonia and spermatocytes. Histological examination of the hCG– group at the FM and NM after 8 weeks revealed that the testes were still immature and contained both spermatogonia and spermatocytes (Figs. 4C, 4D). By contrast, in the hCG+ group, the testes of eels in the FM (Fig. 4E) and NM (Fig. 4F) groups were mature and fully contained spermatozoa after 8 weeks.

### Changes in the GSI and EI values during artificially induced testis development

**Figure 5:**
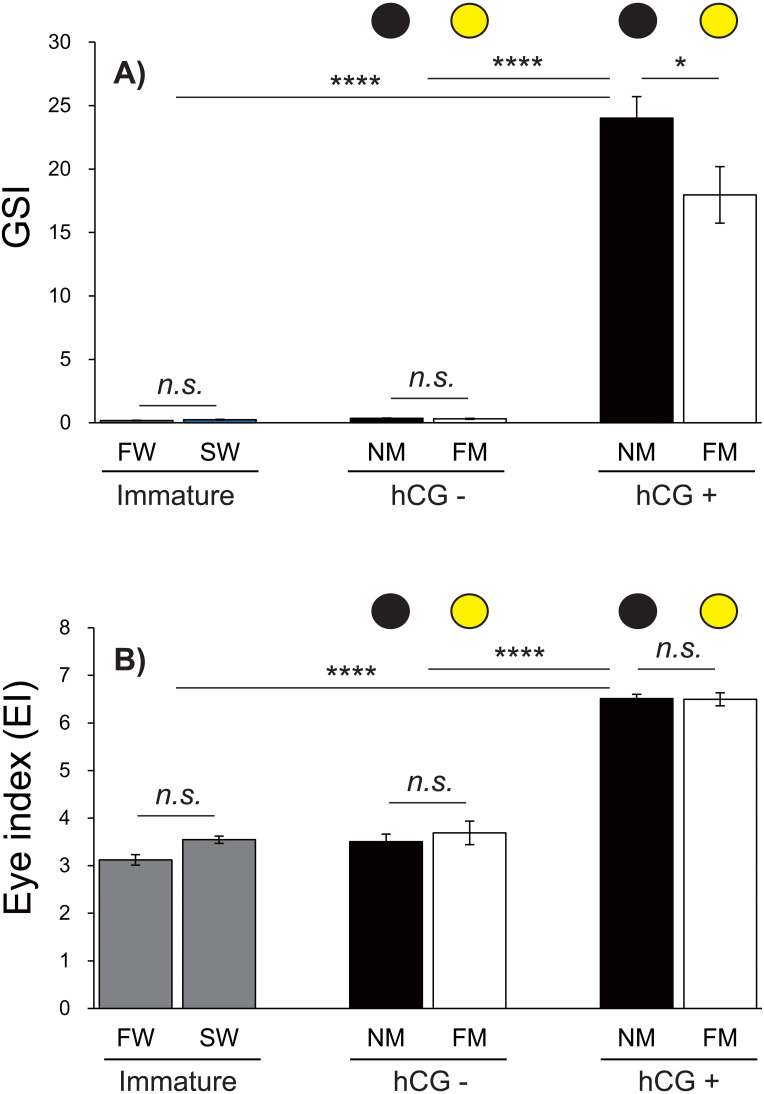
Changes of EI and GSI values. The black and yellow circles represent data from the NM and FM groups, respectively. The GSI (A; *F* = 69.04, *df* = 45) and EI (B; *F* = 130.6, *df* = 45) values shown were determined after FW and SW acclimation, for Japanese eels in the NM and FM groups. In all graphs, the significance levels are as follows: ^∗^*P* < 0.05; ^∗∗∗^*P* < 0.001; ^∗∗∗∗^*P* < 0.0001. The error bars represent the SEMs (*n* = 5–9 fish/treatment).

Changes in the GSI and EI values of male Japanese eels are shown in Fig. 5. The GSI values were low during acclimation to fresh water and sea water. After 8 weeks, no significant differences were observed in the GSI values in the hCG– group at the NM and FM or in the groups acclimated to fresh water (FW) or sea water (SW). The GSI values of the hCG+ group were different in the NM and FM groups (Fig. 5A) (*P* < 0.05).

The EI values were not significantly different between the FW and SW groups. After 8 weeks, the EI values in the hCG– groups at both NM and FM were higher than those in FW and SW (*P* < 0.001). After 8 weeks, the EI values of the hCG– group at FM and NM were significantly lower than those in the hCG+ group (*P* <  0.001), with no significant difference between the NM and FM conditions (Fig. 5B).

**Figure 6:**
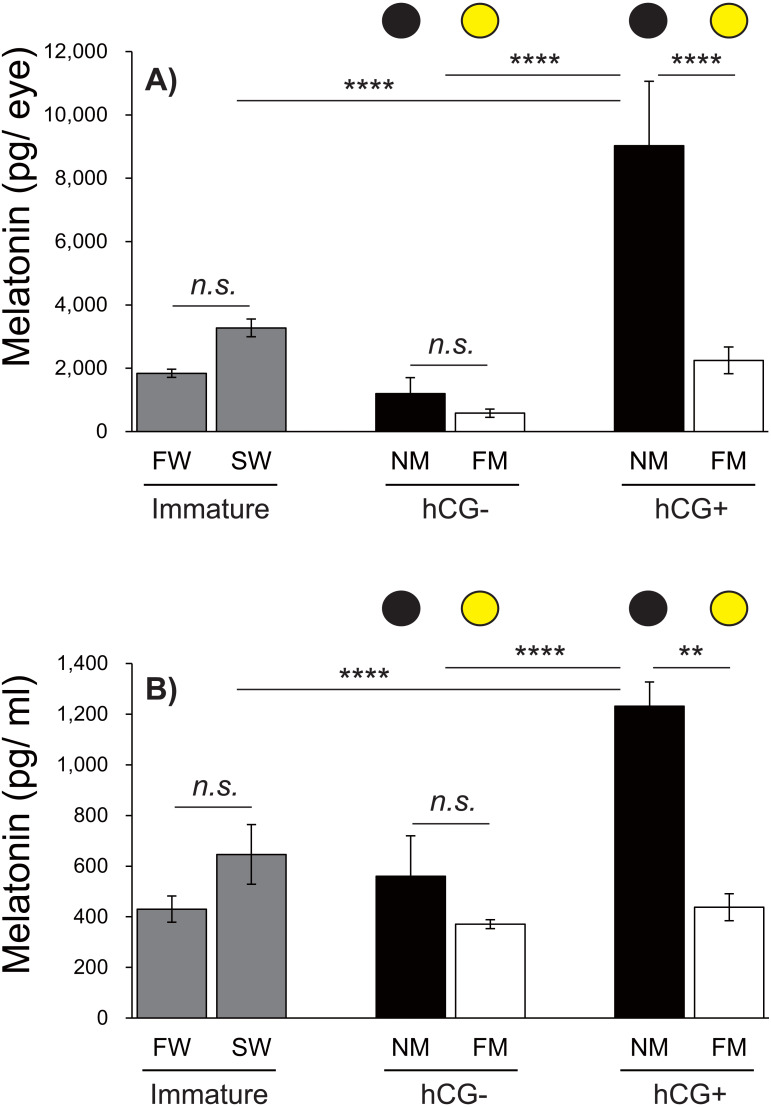
Changes in eye and plasma melatonin levels between Japanese eels exposed to an NM or FM, according to testis development. The black and yellow circles represent eels from the NM and FM groups, respectively. The levels of eye (A; *F* = 14.93, *df* = 42) and plasma melatonin (B; *F* = 16.43, *df* = 43) in hCG+ group after NM exposure were significantly higher than those in the other groups. In all graphs, the significance levels are as follows: ^∗∗^*P* < 0.01; ^∗∗∗∗^*P* < 0.0001. The error bars represent the SEM (*n* = 5–9 fish/treatment).

### Changes in eye and plasma melatonin levels between the NM and FM, according to testis development

Next, we investigated the effects of moonlight on the eye and plasma melatonin levels, according to the sexual maturity of male Japanese eels (Fig. 6). After a 1-week acclimation period in SW, the eye melatonin levels were slightly higher than those in FW, although the difference was not statistically significant. There was no significant difference in eye melatonin levels at NM and FM in the hCG– group at 8 weeks, whereas significantly higher melatonin levels were found at the NM than at the FM in the hCG+ group (*P* <  0.0001). The plasma melatonin levels in the SW group were higher than those in the FW group; however, no significant difference was found. In the hCG– group, the plasma melatonin levels in the NM group were slightly higher than those in the FM group, but the difference was not statistically significant. However, the plasma melatonin levels were significantly higher at NM than at FM in the hCG+ group (*P* <  0.01).

## Discussion

In this study, we showed the plasma melatonin rhythms have similar fluctuation patterns to eye melatonin rhythms. Studies in the sheep *Ovis aries* demonstrated that melatonin produced by the pineal gland was released into the CSF, resulting in approximately 20-fold higher CSF melatonin levels compared with those in the plasma (Skinner & Malpaux, 1999). Although melatonin is also detectable in the retina (Iigo et al., 2007) and in the gastrointestinal tract (Vera et al., 2007), it remains unclear to what extent the melatonin secreted by a non-pineal gland organ contributes to the plasma level. The fluctuation patterns between eye and plasma melatonin in Japanese eel were similar under the 12L12D and DD conditions employed in this study; however, a different fluctuation pattern was observed under the LL condition. These findings imply a partial influence of eye melatonin on plasma melatonin levels, although it is also possible that eye melatonin secreted in the retinas does not directly move to the bloodstream. Unfortunately, this possibility could not be verified in this study because we did not measure melatonin secretion from the pineal gland. Nevertheless, melatonin receptors have been found in the retinas of various vertebrates, including fish, demonstrating a role in dopamine release, horizontal cell sensitivity, and in regulating physiological processes, as detected by electroretinogram findings (Cahill, Grace & Besharse, 1991; Vera et al., 2007). These results certainly suggest that high melatonin concentrations may also play a crucial neuromodulatory role in the retinas in Japanese eels; however, this possibility remains to be verified.

Changes in the amounts of eye melatonin and plasma melatonin were observed in each photoperiod tested in this study. Eye melatonin levels were similar under the 12L12D and SD conditions; however, with a long photoperiod, the melatonin levels peaked at CT4. With respect to plasma, different melatonin-secretion patterns were found between the 12L12D condition and other conditions. With a short photoperiod, melatonin secretion began when the lights were turned off, whereas no difference in the daily amount of melatonin was found in the LD condition. In a study conducted in turkey (*Meleagris gallopavo*), the production period of retinal and pineal melatonin was found to be significantly longer with a short photoperiod than with a regular photoperiod (Zawilska et al., 2006; Zawilska et al., 2007). In particular, both tissues displayed amplitudes in melatonin production during the short photoperiod. These findings imply that melatonin production is correlated with light-exposure time based on the photoperiod. Although it is considered that secretion of high melatonin concentrations by the retinas does not directly affect the plasma concentration—which can explain the different retina and plasma melatonin-fluctuation patterns found with photoperiodic changes—no detailed evidence has been found to support this possibility. Nevertheless, it may be speculated that periodic changes are involved. In general, melatonin signals act collectively as an important endocrine factor in synchronizing the annual reproductive cycle in teleosts (Falcón et al., 2007). However, Japanese eels spawn only once in their lifetime and undergo a photoperiodic change during spawning migration because they migrate from a temperate zone to sub-tropical and tropical zones. Thus, the influence of such photoperiodic changes and/or particular photic signals on eel physiology, and the influence of cumulative changes over the preceding years on synchronizing eel spawning migration should be taken into consideration.

In this study, we investigated changes in melatonin levels under natural moonlight conditions (NM and FM). In the sexually mature, male hCG+ group under an NM, eye melatonin and plasma melatonin levels were significantly higher than those detected in the other treatment groups. These findings imply that the moonlight of the FM partially inhibited melatonin synthesis. A similar pattern of inhibition was found in lunar melatonin rhythms of golden rabbitfish inhabiting a tropical zone based in both *in vivo* and *in vitro* assays (Takemura et al., 2004; Takemura et al., 2006). The plasma melatonin levels of individuals in a tank exposed to the natural light from an FM at midnight were markedly reduced compared with those in a covered tank (Takemura et al., 2004). In numerous previous studies of teleosts, retina melatonin levels exhibited nocturnal patterns that oscillated in a daily/circadian rhythm (Cahill, 1996; Iigo et al., 1997a; Iigo, Tabata & Aida, 1997b; Iigo et al., 2006; Iigo et al., 2007; Rahman et al., 2004; Takeuchi et al., 2014). Similarly, light from an FM led to inhibition of the eye melatonin levels in seagrass rabbitfish (Rahman et al., 2004). These studies in fish that can sense changes in moonlight suggest that FM light can inhibit eye and plasma melatonin. However, in this study, we found no significant difference in eye and plasma melatonin levels between sexually immature eels exposed to an NM or FM.

Anguillids only spawn once in their lifetime. Moreover, sexual maturity occurs only during the spawning migration period. Therefore, the concept of the annual reproductive cycle does not apply for these species. Accordingly, it is likely that eels sense environmental changes that occur repeatedly and use specific environmental signals to initiate reproductive activity without synchronizing their spawning rhythms. In addition, Sébert et al. (2008) revealed that 5-month melatonin implantation increased the mRNA expression of brain tyrosine hydroxylase in sexually immature female European eels and inhibited the synthesis and release of pituitary gonadotropin (follicle-stimulating hormoneβ and luteinizing hormoneβ). These findings raised the possibility that melatonin may have negative effects during the early stages of sexual maturation and puberty. Nonetheless, these results could not confirm the role of melatonin during the spawning period because sexually immature females were used in the experiments. A notable difference in melatonin secretion was found in the present study between sexually mature (hCG+ group) and immature (hCG– group) eels. This finding suggests that changes in melatonin levels due to moonlight may be involved in spawning, at least partially during the spawning period of sexually mature eels, despite the negative or negligible influence of melatonin on the brain–pituitary–gonad axis during the early stages of sexual maturation (puberty). The spawning period of Japanese eels is estimated to occur toward the last days of the lunar month (Tsukamoto et al., 2011), which appears to be correlated with melatonin production. However, it remains unclear whether moonlight directly regulates melatonin, or if moonlight simply has a promoting effect following the activation of other endocrine factors during sexual maturation.

Japanese eels exhibit diel vertical migration with repetitive rises and falls based on the day–night cycle during spawning migration (nighttime: 100–500 m; daytime: 500–800 m) (Chow et al., 2015; Manabe et al., 2011). This may be attributed to the eel moving deeper into the water to continuously be in relatively dark conditions, which likely helps to increases melatonin secretion during sexual maturation (spawning migration). In addition, previous reports have shown interesting results regarding the locomotor activity of Japanese eels in relation to the lunar cycle. Monitoring eel behavior during spawning migration through short-term tracking with an ultrasonic transmitter revealed that the rise to the upper mesopelagic zone under an NM or a very small moon after sunset was contrasted by the trend to fall deeper as the moon grew in size, followed by another rise to the upper mesopelagic zone toward the moon set (Chow et al., 2015). These findings indicate a direct correlation between the locomotor activities of eels and the sunlight or moonlight, in which eels sense the moonlight through their retinas, while melatonin plays a crucial role in promoting their locomotor activities.

In this study, we examined changes in the ocular and plasma melatonin levels in Japanese eels according to changes in moonlight and during the circadian cycle, photoperiod, and spawning period. Our findings suggest that melatonin signaling results from the eels sensing photic cues to regulate the physiology. Furthermore, eels are considered to sense the moonlight during the spawning period and use it as a signal for oviposition (Tesch, 1978). According to Ikegami et al. (2014), fish species in tropical regions reside in a location where temperature or photoperiod changes are relatively less frequent (*i.e.,* a relatively more stable aquatic environment) compared to fish species residing in temperate regions. Consequently, the moonlight signal promotes synchronization during several biological and physiological processes.

The spawning period of Japanese eel is estimated to occur around the NM (Tsukamoto et al., 2003). These results suggest that lunar signals may serve as a key link in regulating endocrine secretion in anguillids. Thus, further studies are need to evaluate the biological activities of melatonin mediated by photic signals, as well as to identify the basic melatonin-secretion patterns that depend on nocturnal eel behaviors. Anguillids may provide a useful model for addressing even more intriguing topics given the limited knowledge of the endocrinology and ecology of these species, despite presumptions regarding their spawning sites or reports on their spawning ecology (Aoyama et al., 2014; Tsukamoto et al., 2003).

## Conclusions

Both eye and plasma melatonin levels were regulated by daylight cycling and circadian oscillations, and melatonin was inhibited under natural moonlight exposure in sexually mature Japanese male eels. Thus, photic cues from daylight and nocturnal moonlight may correlate with nocturnal behavioral responses, including testis development and spawning during the NM period.

## Supplemental Information

10.7717/peerj.12289/supp-1 Supplemental Information 1 Raw data

## Additional Information and Declarations

### Data Availability

The following information was supplied regarding data availability: The raw data are available as a Supplemental File.
